# Supplementary material for: HIVprotI: an integrated web based platform for prediction and design of HIV proteins inhibitors
Source: J Cheminform. 2018 Mar 9;10:12. doi: 10.1186/s13321-018-0266-y (PMC5845081; doi:10.1186/s13321-018-0266-y)
Supplement: Supplementary file 2 — Additional file 2. Source code of HIVProtI web server. [file 13321_2018_266_MOESM2_ESM.zip › HIVprotI_Source-code/hivprotI/weka-3-6-6/doc/weka/core/matrix/IntVector.html]

IntVector


---


|  |  |  |
| --- | --- | --- |
| **Overview** \| **Package** \| **Class** \| **Tree** \| **Deprecated** \| **Index** \| **Help** \| **Weka's home** | |  |
| **PREV CLASS**   **NEXT CLASS** | **FRAMES**    **NO FRAMES**     **All Classes** |  |
| SUMMARY: NESTED \| FIELD \| CONSTR \| METHOD | DETAIL: FIELD \| CONSTR \| METHOD |  |


---


## weka.core.matrix Class IntVector

```
java.lang.Object
  weka.core.matrix.IntVector
```

**All Implemented Interfaces:**: java.lang.Cloneable, RevisionHandler

---

``` public class IntVector extends java.lang.Object implements java.lang.Cloneable, RevisionHandler ```

A vector specialized on integers.

**Version:**
:   $Revision: 1.4 $

**Author:**
:   Yong Wang

---

**Constructor Summary**

`IntVector()`
  
          Constructs a null vector.

`IntVector(int n)`
  
          Constructs an n-vector of zeros.

`IntVector(int[] v)`
  
          Constructs a vector given an int array

`IntVector(int n,
int s)`
  
          Constructs an n-vector of a constant


| **Method Summary** | |
| --- | --- |
| `int` | `capacity()`             Returns the capacity of the vector |
| `java.lang.Object` | `clone()`             Clones the IntVector object. |
| `IntVector` | `copy()`             Makes a deep copy of the vector |
| `int` | `get(int i)`             Gets the value of an element. |
| `int[]` | `getArray()`             Access the internal one-dimensional array. |
| `int[]` | `getArrayCopy()`             Returns a copy of the internal one-dimensional array. |
| `java.lang.String` | `getRevision()`             Returns the revision string. |
| `boolean` | `isEmpty()`             Returns true if the vector is empty |
| `static void` | `main(java.lang.String[] args)`             Tests the IntVector class |
| `static IntVector` | `seq(int i0, int i1)`             Generates an IntVector that stores all integers inclusively between two integers. |
| `void` | `set(int s)`             Sets the value of an element. |
| `void` | `set(int i, int s)`             Sets a single element. |
| `void` | `set(int i0, int i1, int[] v, int j0)`             Sets the values of elements from an int array. |
| `void` | `set(int i0, int i1, IntVector v, int j0)`             Sets the values of elements from another IntVector. |
| `void` | `set(IntVector v)`             Sets the values of elements from another IntVector. |
| `void` | `setCapacity(int capacity)`             Sets the capacity of the vector |
| `void` | `setSize(int size)`             Sets the size of the vector. |
| `void` | `shift(int i, int j)`             Shifts an element to another position. |
| `void` | `shiftToEnd(int j)`             Shifts an element to the end of the vector. |
| `int` | `size()`             Gets the size of the vector. |
| `void` | `sort()`             Sorts the elements in place |
| `IntVector` | `subvector(int i0, int i1)`             Returns a subvector. |
| `IntVector` | `subvector(IntVector index)`             Returns a subvector as indexed by an IntVector. |
| `void` | `swap(int i, int j)`             Swaps the values stored at i and j |
| `java.lang.String` | `toString()`             Converts the IntVecor to a string |
| `java.lang.String` | `toString(int digits, boolean trailing)`             Convert the IntVecor to a string |

**Methods inherited from class java.lang.Object**

`equals, getClass, hashCode, notify, notifyAll, wait, wait, wait`

**Constructor Detail**

### IntVector

```
public IntVector()
```

:   Constructs a null vector.

---


### IntVector

```
public IntVector(int n)
```

:   Constructs an n-vector of zeros.

    **Parameters:**: `n` - Length.

---


### IntVector

```
public IntVector(int n,
                 int s)
```

:   Constructs an n-vector of a constant

    **Parameters:**: `n` - Length.

---


### IntVector

```
public IntVector(int[] v)
```

:   Constructs a vector given an int array

    **Parameters:**: `v` - the int array


**Method Detail**

### size

```
public int size()
```

:   Gets the size of the vector.

    :   **Returns:**: Size.

---


### setSize

```
public void setSize(int size)
```

:   Sets the size of the vector. The provided size can't be greater than
    the capacity of the vector.

    :   **Parameters:**: `size` - the new Size.

---


### set

```
public void set(int s)
```

:   Sets the value of an element.

    :   **Parameters:**: `s` - the value for the element

---


### set

```
public void set(int i0,
                int i1,
                int[] v,
                int j0)
```

:   Sets the values of elements from an int array.

    :   **Parameters:**: `i0` - the index of the first element: `i1` - the index of the last element: `v` - the int array that stores the values: `j0` - the index of the first element in the int array

---


### set

```
public void set(int i0,
                int i1,
                IntVector v,
                int j0)
```

:   Sets the values of elements from another IntVector.

    :   **Parameters:**: `i0` - the index of the first element: `i1` - the index of the last element: `v` - the IntVector that stores the values: `j0` - the index of the first element in the IntVector

---


### set

```
public void set(IntVector v)
```

:   Sets the values of elements from another IntVector.

    :   **Parameters:**: `v` - the IntVector that stores the values

---


### seq

```
public static IntVector seq(int i0,
                            int i1)
```

:   Generates an IntVector that stores all integers inclusively between
    two integers.

    :   **Parameters:**: `i0` - the first integer: `i1` - the second integer

---


### getArray

```
public int[] getArray()
```

:   Access the internal one-dimensional array.

    :   **Returns:**: Pointer to the one-dimensional array of vector elements.

---


### sort

```
public void sort()
```

:   Sorts the elements in place

---


### getArrayCopy

```
public int[] getArrayCopy()
```

:   Returns a copy of the internal one-dimensional array.

    :   **Returns:**: One-dimensional array copy of vector elements.

---


### capacity

```
public int capacity()
```

:   Returns the capacity of the vector

---


### setCapacity

```
public void setCapacity(int capacity)
```

:   Sets the capacity of the vector

    :   **Parameters:**: `capacity` - the new capacity of the vector

---


### set

```
public void set(int i,
                int s)
```

:   Sets a single element.

    :   **Parameters:**: `i` - the index of the element: `s` - the new value

---


### get

```
public int get(int i)
```

:   Gets the value of an element.

    :   **Parameters:**: `i` - the index of the element **Returns:**: the value of the element

---


### copy

```
public IntVector copy()
```

:   Makes a deep copy of the vector

---


### clone

```
public java.lang.Object clone()
```

:   Clones the IntVector object.

    :   **Overrides:**: `clone` in class `java.lang.Object`

---


### subvector

```
public IntVector subvector(int i0,
                           int i1)
```

:   Returns a subvector.

    :   **Parameters:**: `i0` - the index of the first element: `i1` - the index of the last element **Returns:**: the subvector

---


### subvector

```
public IntVector subvector(IntVector index)
```

:   Returns a subvector as indexed by an IntVector.

    :   **Parameters:**: `index` - the index **Returns:**: the subvector

---


### swap

```
public void swap(int i,
                 int j)
```

:   Swaps the values stored at i and j

    :   **Parameters:**: `i` - the index i: `j` - the index j

---


### shift

```
public void shift(int i,
                  int j)
```

:   Shifts an element to another position. Elements between them are
    shifted one position left.

    :   **Parameters:**: `i` - the index of the element: `j` - the index of the new position

---


### shiftToEnd

```
public void shiftToEnd(int j)
```

:   Shifts an element to the end of the vector. Elements between them are
    shifted one position left.

    :   **Parameters:**: `j` - the index of the element

---


### isEmpty

```
public boolean isEmpty()
```

:   Returns true if the vector is empty

---


### toString

```
public java.lang.String toString()
```

:   Converts the IntVecor to a string

    :   **Overrides:**: `toString` in class `java.lang.Object`

---


### toString

```
public java.lang.String toString(int digits,
                                 boolean trailing)
```

:   Convert the IntVecor to a string

    :   **Parameters:**: `digits` - number of digits to be shown: `trailing` - true if trailing zeros are to be shown

---


### getRevision

```
public java.lang.String getRevision()
```

:   Returns the revision string.

    :   **Specified by:**: `getRevision` in interface `RevisionHandler`
    :   **Returns:**: the revision

---


### main

```
public static void main(java.lang.String[] args)
```

:   Tests the IntVector class


---


|  |  |  |
| --- | --- | --- |
| **Overview** \| **Package** \| **Class** \| **Tree** \| **Deprecated** \| **Index** \| **Help** \| **Weka's home** | |  |
| **PREV CLASS**   **NEXT CLASS** | **FRAMES**    **NO FRAMES**     **All Classes** |  |
| SUMMARY: NESTED \| FIELD \| CONSTR \| METHOD | DETAIL: FIELD \| CONSTR \| METHOD |  |


---
